# Age-period-cohort analysis of depression trends: are depressive symptoms increasing across generations in Germany?

**DOI:** 10.1007/s10433-022-00732-z

**Published:** 2022-10-06

**Authors:** Johannes Beller

**Affiliations:** Hannover Medical School, Center for Public Health and Health Care, Medical Sociology Unit, Carl-Neuberg-Str. 1, 30625 Hannover, Germany

**Keywords:** Depression, Mental health, Trend, Age-period-cohort, Generation

## Abstract

Several studies have examined trends in depression, but only few have explicitly considered possible generational differences. I examined changes in the burden of depressive symptoms between 2002 and 2017 according to age, time period and birth cohort in Germany. I used population-based data drawn from the German Aging Survey (*N* = 33,723, 54% female, ages 40 +) from 2002, 2008, 2011, 2014, and 2017. Depressive symptoms were measured via the CES-D 15. Hierarchical age-period-cohort models were used to examine trends in depression. I found that depressive symptoms changed across age, time period and birth cohorts. While there was a general decrease across time periods, strong evidence for a U-shaped cohort effect was also found: Younger generations, beginning with cohorts born after the World War II, increasingly report more depressive symptoms than older generations. This U-shaped cohort trend appeared most pronounced for the somatic symptoms subscale. Contrarily, only minimal cohort differences were found regarding the positive affect subscale. Therefore, depressive symptoms, and especially somatic symptoms, seem to increase in more recent birth cohorts in Germany, who might thus be at risk to experience more mental health problems in the future. Potential reasons for these trends and the generalizability of the results to other countries should be investigated by future studies.

## Introduction

Depression is seen as the result of a complex relationship between personal and contextual factors, encompassing affective-cognitive as well as somatic symptoms (Goldberg, 2006). Depression poses a significant burden to global population health (James et al., 2018): In addition to being a common and severe disorder itself, depression and depressive symptoms have also been shown to be major predictors of other health-related outcomes including cardiovascular disease, early retirement, dementia, and mortality (Byers & Yaffe, 2011; Gilman et al., 2017; Karpansalo, 2005; Rice et al., 2011; Thom et al., 2019). Consequently, depression is also one of the leading causes of life years lost due to ill-health, disability and early death (Kassebaum et al., 2016; Vigo et al., 2016).

Therefore, studies on trends in depressive symptoms are needed (Bretschneider et al., 2018; Thomson & Katikireddi, 2018). Changes in depressive symptoms over time can occur according to three time-related variables: age, time period and birth cohort (Y. Yang & Land, 2013). Regarding age, depressive symptoms could change over time simply due to population aging processes and the accompanying changes in mental health risks such as increased social isolation in old age (Beller & Wagner, 2018; Cacioppo et al., 2010). In accordance with this possibility, depressive symptoms have been shown to increase in old age (Bell, 2014; Beller et al., 2020; Sutin et al., 2013). Depressive symptoms might also vary with the time period, which corresponds to the calendar years depressive symptoms are measured. As one example, recent large-scale changes in risk factors for depressive symptoms might have also affected depressive symptoms in the general population of developed countries, such as increased financial difficulties during the 2008 financial crisis, increased sedentary lifestyle, or advances in mental health treatments and health care access (e.g., Angermeyer et al., 2014; Lee et al., 2010; Pescosolido et al., 2021; L. Yang et al., 2019). Finally, depressive symptoms might also change over time due to birth cohorts. This refers to generational effects that affect groups born within a particular time period that, because they are born in a similar time, share similar socio-historical experiences (Colman & Ataullahjan, 2010). These shared socio-historical experiences throughout the life course might affect the risk of developing depressive symptoms in specific birth cohorts. As a prominent example, experiencing the deprivation following World War II in Germany might have increased the lifelong risk to experience depressive symptoms in the respective birth cohorts of Germans (Stephan et al., 2019). Thus, trends in depression can occur due to age, period, and cohort effects.

Several studies have analyzed trends in depression, but recently only few studies have explicitly included the birth cohort as one of the potentially important contextual factors in mental health development, with contradicting results. For example, Twenge and colleagues used hierarchical age-period-cohort (HAPC) models to analyze trends in diverse indicators of mood disorder among US youth and adults (Twenge et al., 2019). They found that trends were primarily due to cohort differences, with younger generations exhibiting increasing levels of psychological distress. Similarly, Sullivan and colleagues (Sullivan et al., 2020) used a modified version of the 8-item Center for Epidemiological Studies Depression Scale (CES-D) and found that the depressive burden of US older adults decreased across birth cohorts. In contrast, Keyes and colleagues (2019) recently analyzed trends in depressive symptoms of US students of the 8th, 10th, and 12th grade from 1991 to 2018. Here, depression was measured as a continuous construct via self-report as a sum score of four depressive symptoms. The authors found that depressive symptoms increased in their samples over time, especially among girls. However, cohort effects were found to be minimal at best. In a European context, Spiers and colleagues (2011) analyzed age, period and birth cohort differences in the prevalence of common mental disorder. Using an English sample, they found little evidence for changes in common mental disorder over time. Contrasting with these results, Bramajo (2022) recently analyzed trends in depression in six European countries, including Germany. Using age-period-cohort methods, they found increases in the prevalence of depression among younger born cohorts, especially among men. Hence, studies on trends in depression disagree regarding the importance of the birth cohort: While some studies find strong birth cohort differences in depressive symptoms, others fail to replicate this finding. Additionally, although depression is seen as encompassing heterogeneous symptoms from affective as well as somatic domains, studies are lacking that examine how sub-dimensions of depressive symptoms vary over time (den Hollander-Gijsman et al., 2012). Furthermore, previous studies have been limited geographically, in that they mostly stem from samples in the USA. Thus, more research on generational differences in depressive symptoms is needed.

The current study aims to address these issues. It contributes to the literature by examining age-period-cohort differences in depressive symptoms between 2002 and 2017, explicitly also considering sub-dimensions of depression and utilizing a large population-based German sample (*N* = 33,723). Thereby the current study clarifies how depressive symptoms and their affective and somatic sub-dimensions have changed over time in Germany and the degree to which cohort effects/generational differences might explain these trends.

## Methods

### Sample

Data were drawn from public releases of the German Aging Survey (Engstler & Hameister, 2021a, 2021b, 2021c, 2021d; Klaus et al., 2017; Vogel et al., 2021). The German Aging Survey (Deutscher Alterssurvey; DEAS) is a cohort-sequential longitudinal, population-based study on Germans aged 40 years and older that is provided by the Research Data Center of the German Center of Gerontology (Klaus et al., 2017; Mahne et al., 2020; Motel-Klingebiel et al., 2016). For the German Aging Survey, participants are drawn randomly by probability sampling in 1996, 2002, 2008 and 2014. Additionally, participants from previous waves are re-contacted in 2002, 2008, 2011, 2014 and 2017. All interviews are conducted face-to-face in the participant’s residence. All procedures are in accordance with German law and the ethical standards of the 1964 Helsinki declaration and its later amendments. I used data from all participants in 2002, 2008, 2011, 2014, and 2017 who filled out a drop-off questionnaire. The 2002 wave was the first one to include the measure of depressive symptoms. After excluding participants with missing values listwise (about 2.6% of the sample), a final sample with *N* = 33,723 participants resulted (*N*_2002_ = 4298; *N*_2008_ = 8027; *N*_2011_ = 4765; *N*_2014_ = 10,113; *N*_2017_ = 6520).

### Measures

Depressive symptoms were measured with the 15-item version of the Center of Epidemiologic Studies Depression Scale (CES-D 15) at all time points (Radloff, 1977). Multiple studies have demonstrated the scale’s validity and reliability to measure depressive symptoms in different cultures and throughout the lifespan (e.g., Karim et al., 2015; Kliem et al., 2020; Vilagut et al., 2016). The CES-D 15 measures the frequency of depressive symptoms in the week prior to the interview. Participants were asked whether they (1) “were bothered”, (2) “could not shake off the blues”, (3) “had trouble concentrating”, (4) “felt depressed”, (5) “felt that everything was an effort”, (6) “thought that their life had been a failure”, (7) “felt fearful”, and (8) “slept restlessly”, (9, reverse scored) “were happy”, (10) “talked less than usual”, (11) “felt lonely”, (12, reverse scored) “enjoyed their life”, (13) “felt sad”, (14) “felt that people dislike them”, and (15) “could not get going”. Participants could choose to respond with one of four response options ranging from “none or almost none of the time” (score 0) to “all or almost all of the time” (score 3). In accordance with psychometric evidence, a dimensional mean depressive symptoms score was calculated as the mean of all responses ranging from 0 to 3 (Liu, 2016). Additionally, three subscales of the CES-D, as reported in the literature, were constructed via mean scores also ranging from 0 to 3 (the fourth sub-scale could not be constructed because half of the items were missing from the 15-item version of the CES-D): Negative Affect (items 2, 4, 6, 7, 11, 13), Positive Affect (items 9, 12), and Somatic Symptoms (items 1, 3, 5, 8, 10, 15). In the current study, reliability of the CES-D 15 mean score was acceptable (Cronbach’s *α* = 0.76).

### Data analysis

First, descriptive statistics of all variables across time periods are reported in the results section. Then, to separate the effects of age, time period, and birth cohort, I performed hierarchical age-period-cohort analyses (HAPC; Y. Yang & Land, 2013). The fundamental difficulty in separating age, period and cohort effects is their perfect linear relationship, i.e., age = period-cohort, because of which regular statistical methods such as linear regression analysis cannot be used. To separate age, period and cohort effects, certain assumptions must be made, which are determined by the specific age-period-cohort model one employs. In their HAPC model, Yang and Land (2013) proposed using multilevel models to estimate age-period-cohort effects, with age being assumed to be an individual fixed predictor, nested into time periods and birth cohorts on the second level with random intercepts. Additionally, age is typically used as a continuous variable, whereas time periods and birth cohorts are used as categorical variables, with cohorts being typically grouped into five-year intervals. In line with the estimation of multilevel models, HAPC models are estimated in a two-step procedure in which first the effect of age is calculated, and then, depending on the resulting residuals, the remaining variance is allocated to period and cohort differences (Luo & Hodges, 2020). Thus, in HAPC analysis it is typically assumed that there is a prominent age-effect to which period and cohort differences are subordinated. It is also typically assumed that there are only negligible intra-cohort differences within the 5-year cohort groups and that there are no interaction effects between age, period and cohort. Furthermore, extensive simulation analyses have shown that HAPC models might underestimate cohort effects and might provide inaccurate results when strictly linear cohort effects are present, which, however, seems to be exceedingly rare in empirical research, as argued by proponents of HAPC methodology (Bell & Jones, 2018; Fosse & Winship, 2019; Fu, 2018; Luo & Hodges, 2020; Masters & Powers, 2020; Reither et al., 2015). Notwithstanding the theoretical differences, agreement between the HAPC method and other analysis options is reported to be good (Masters & Powers, 2020).

Following the recommendations of Yang and Land (2013), I estimated these HAPC models with individuals nested in birth cohort groups and time periods, allowing mean levels of depressive symptoms to vary across time periods and birth cohorts. For all HAPC analyses, the depressive symptom scores were z-scaled such that they had a mean of 0 and a standard deviation of 1, in order to improve the interpretability and comparability of results. As such the HAPC results can be interpreted similar to standardized mean differences, e.g., with a value of 0.5 denoting a change of half a standard deviation in depressive symptoms in the general population. Hierarchical age-period-cohort models have been successfully used to examine trends in multiple areas of science, including trends on health (Beller & Epping, 2020; Diouf et al., 2010; Twenge et al., 2019). Age, period and cohort effects can provide evidence on large-scale trends, but, as in regular trend analyses, determining the causal influences generating these trends remains difficult (Bell, 2020). According to the Akaike Information Criterion (AIC), the multilevel model that included both random effects of time period and birth cohort was the most appropriate for the data and is hence used in the current study (Hierarchical Age-Period Model: AIC = 35,711.18; Hierarchical Age-Cohort Model: AIC = 35,659.42; Hierarchical Age-Period-Cohort Model: AIC = 35,613.99). As the results were similar for both genders, the combined analysis is mainly presented in the results section; gender-specific results are, however, also reported. Additionally, several sensitivity analyses are reported in Appendix (an HAPC analysis including only baseline participants and an HAPC analysis using alternative model specifications). All statistical analyses were performed with R.

## Results

Overall, participants were on average 64.15 (*SD* = 11.63) years old, with 50% being female. An average CES-D 15 mean score of 0.45 (scale from 0 to 3; *SD* = 0.41) was found. As depicted in Table 1, on a descriptive level, overall CES-D 15 scores decreased slightly across time (descriptive figures can be found in Appendix Figs. 5 and 6).

**Table 1:** Descriptive statistics across time periods

	2002		2008		2011		2014		2017	
Variable	M/%	SD	M/%	SD	M/%	SD	M/%	SD	M/%	SD
Depressive Symptoms	0.50	0.44	0.43	0.41	0.44	0.41	0.46	0.41	0.44	0.40
Depressed Affect	0.28	0.45	0.23	0.42	0.22	0.40	0.23	0.40	0.22	0.39
Positive Affect	1.31	0.86	1.18	0.89	1.19	0.89	1.18	0.88	1.15	0.87
Somatic Symptoms	0.50	0.51	0.43	0.48	0.46	0.49	0.49	0.49	0.48	0.47
Age	61.75	12.00	62.48	11.90	65.19	11.02	64.24	11.56	66.88	10.89
Gender	49%	–	49%	–	50%	50%	50%	–	50%	–

*N* = Sample size;* M* = Mean; 95%-*CI* = 95%-confidence interval

### Overall depressive symptoms

**Fig. 1:**
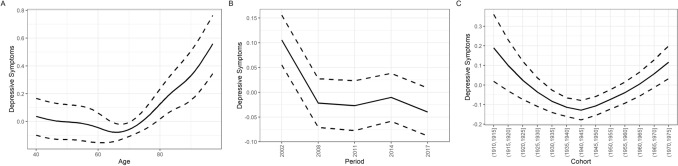
Age, period, and cohort differences (z-standardized) in depressive symptoms according to the HAPC model. Panel A depicts the predicted value of depressive symptoms across age; panel B depicts the predicted value of depressive symptoms across time periods; panel C depicts the predicted value of depressive symptoms across birth cohorts. Dashed lines indicate the 95% confidence intervals

**Fig. 2:**
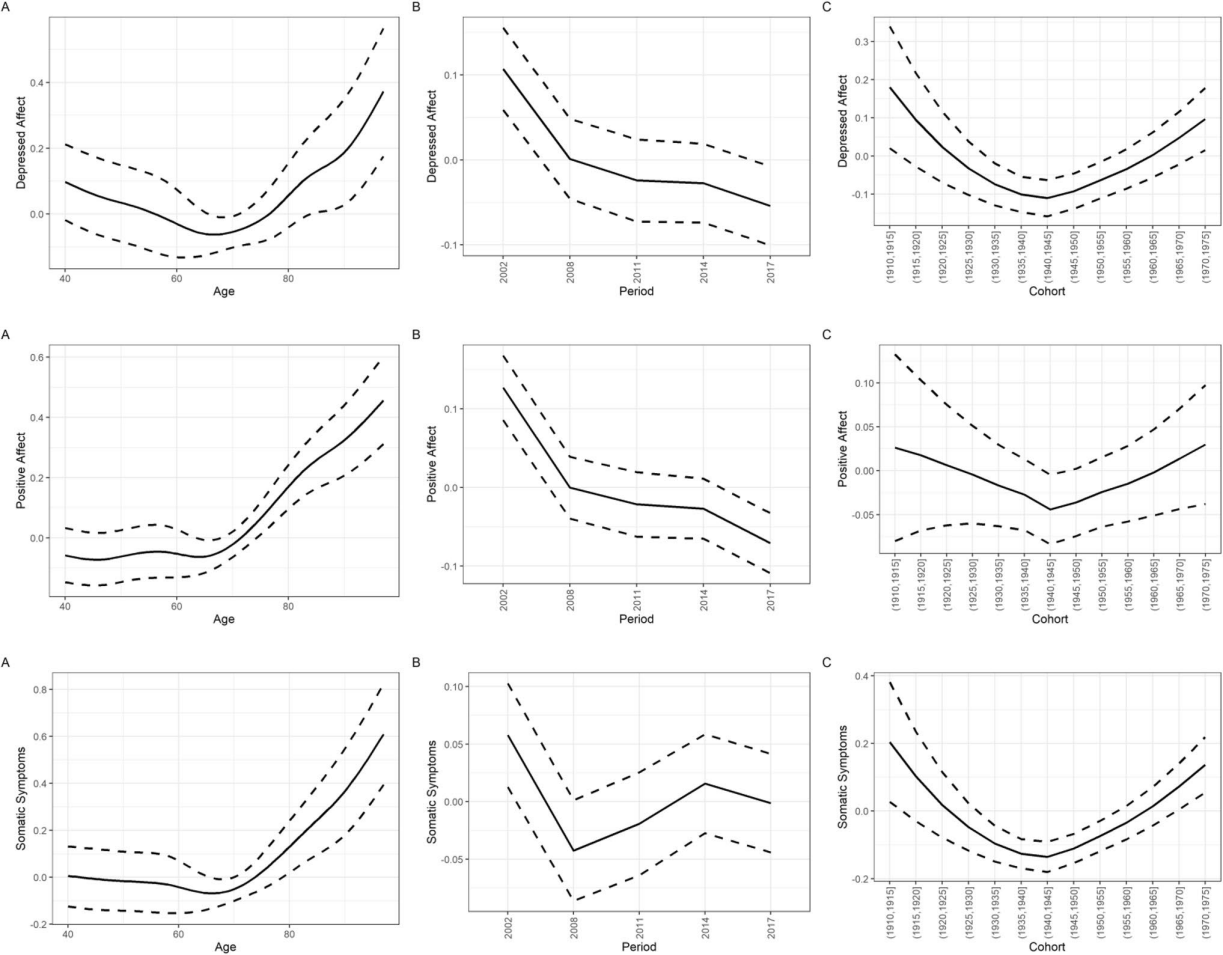
Age, period, and cohort differences (z-standardized) in depressed affect, positive affect and somatic symptoms according to the HAPC model. The upper row depicts the results regarding depressed affect; the middle row depicts the results regarding positive affect; the lower row depicts the results regarding somatic symptoms. Panel A depicts the predicted value of depressed affect, positive affect and somatic symptoms across age; panel B depicts the predicted value of depressed affect, positive affect and somatic symptoms across time periods; panel C depicts the predicted value of depressed affect, positive affect and somatic symptoms across birth cohorts. Dashed lines indicate the 95% confidence intervals

**Fig. 3:**
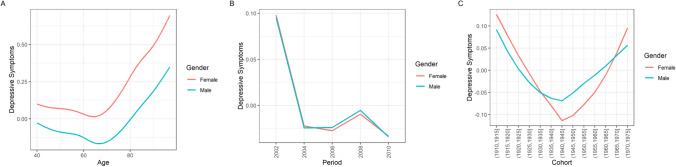
Age, period, and cohort differences (z-standardized) in depressive symptoms stratified by gender according to the HAPC model. Panel A depicts the predicted value of depressive symptoms across age; panel B depicts the predicted value of depressive symptoms across time periods; panel C depicts the predicted value of depressive symptoms across birth cohorts

Next, I used HAPC analysis to disentangle the observed descriptive changes in depressive symptoms by age, time period, and birth cohort effects (please see the Appendix Tables 2, 3, 4 and 5 for the full numeric regression results). Regarding overall depressive symptoms, intercepts varied mostly due to birth cohort (*SD* = 0.11; Fig. 1, panel C), and to a lesser degree due to time period (*SD* = 0.07; Fig. 1, upper row, panel B). Depressive symptoms increased over age, after the age of about 65, in a linear way. After controlling for age and cohort effects, depressive symptoms decreased across time points. Birth cohorts showed a U-shaped pattern, with cohorts born around 1930 until 1950 exhibiting less overall depressive symptoms than earlier and later born cohorts. As seen in Fig. 3, trends were similar for men and women. Maximal inter-cohort differences (about *d* = 0.2) were similar in size to the gender difference in depressive symptoms across age (Fig. 2).

### Subscales of depressed affect, positive affect, and somatic symptoms

**Fig. 4:**
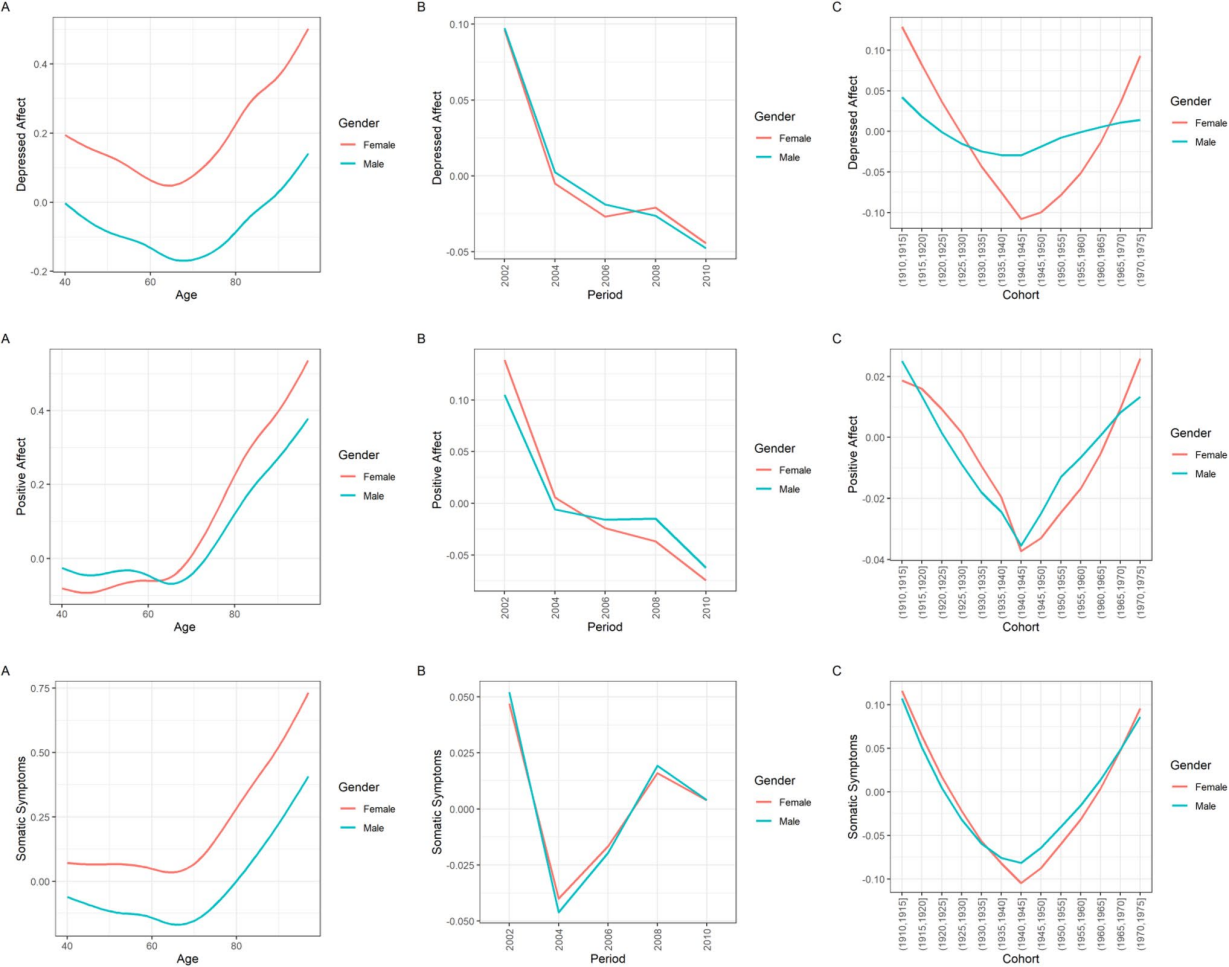
Age, period, and cohort differences (z-standardized) in depressed affect, positive affect and somatic symptoms stratified by gender according to the HAPC model. The upper row depicts the results regarding depressed affect; the middle row depicts the results regarding positive affect; the lower row depicts the results regarding somatic symptoms. Panel A depicts the predicted value of depressed affect, positive affect and somatic symptoms across age; panel B depicts the predicted value of depressed affect, positive affect and somatic symptoms across time periods; panel C depicts the predicted value of depressed affect, positive affect and somatic symptoms across birth cohorts

Results regarding the subscales of depressed affect, positive affect and somatic symptoms were in general similar to the results of the overall scale. However, birth cohort differences were largest regarding the somatic symptoms subscale, followed by the depressed affect subscale and, with a minimal effect size, positive affect. Contrarily, time period differences appeared to be largest regarding the positive affect subscale, followed by the depressed affect subscale and, with the smallest effect size, somatic symptoms. As seen in Fig. 4, trends in subscales of depressed effect were also similar for men and women. Only in the case of depressed affect were the inter-cohort differences notably larger in women as compared to men. Again, similar to the global depressive symptoms score, maximal inter-cohort differences (about *d* = 0.2) were similar in size to the gender difference in the subscales across age.

Regarding the depressed affect subscale, intercepts varied also mostly due to birth cohort (*SD* = 0.10; Fig. 1, panel C), and to a lesser degree due to time period (*SD* = 0.07; Fig. 1, upper row, panel B). Depressed affect increased over age, after the age of about 65, in a linear way. After controlling for age and cohort effects, depressive symptoms decreased across time points. Birth cohorts again showed a U-shaped pattern, with cohorts born around 1930 until 1950 exhibiting less depressed affect symptoms than earlier or later born cohorts.

Regarding the positive affect subscale, intercepts varied only slightly due to both birth cohort (*SD* = 0.06; Fig. 1, panel C), and time period (*SD* = 0.08; Fig. 1, upper row, panel B). The positive affect mean score again increased over age, after the age of about 65, in a linear way. After controlling for age and cohort effects, depressive symptoms decreased across time points. Birth cohorts showed a marginally U-shaped pattern, with cohorts born around 1930 until 1950 exhibiting slightly less (lack of) positive affect symptoms than earlier or later born cohorts.

Regarding the somatic symptoms subscale, intercepts varied mostly due to birth cohort (*SD* = 0.12; Fig. 1, panel C), and to a lesser degree due to time period (*SD* = 0.05; Fig. 1, upper row, panel B). Somatic symptoms increased over age, after the age of about 65, in a linear way. After controlling for age and cohort effects, depressive symptoms decreased across time points. Here, birth cohorts also showed a U-shaped pattern, with cohorts born around 1930 until 1950 exhibiting less overall depressive symptoms than earlier or later born cohorts.

## Discussion

I examined age-period-cohort trends in depressive symptoms in Germany and found that depressive symptoms descriptively decreased across time periods. However, I also found evidence for a U-shaped cohort effect: Younger generations, beginning with cohorts born after the World War II, reported more depressive symptoms than older generations. Although strict conclusions due to overlapping confidence intervals between subscales are not possible, this U-shaped cohort trend appeared most pronounced for somatic symptoms.

Previous studies in US and European samples had provided conflicting evidence regarding the existence of a cohort effect in trends in depressive symptoms, with some providing support for a cohort effect (Bramajo, 2022; Sullivan et al., 2020; Twenge et al., 2019), and others providing no support for it (Keyes et al., 2019; Spiers et al., 2011). As I found evidence for a general cohort effect in Germany, the current study supports the existence of a cohort effect in depression trends. Going beyond previous studies, I was able to also study trends in sub-dimensions of depression. Here, I found that the strongest cohort differences were observed in somatic symptoms, while cohort differences in (lack of) positive affect can only be described as minimal (however, it must be noted that the confidence intervals between subscales were overlapping and thus these results must be taken as preliminary). Furthermore, the effect sizes of these inter-cohort differences were substantial: Comparing the cohorts born in 1940–1945 and 1970–1975 results in a difference of about *d* = 0.2, which roughly corresponds to the often-analyzed gender differences in depressive symptoms observed in this study and others (Salk et al., 2017). Hence, one potential explanation for previous contrasting results is that the extent of cohort differences in depressive symptoms differs depending on their specific latent content. If substantiated, these results are alarming from a public mental health perspective (Eaton & Fallin, 2019). As mental health problems are often chronic, future generations might experience more long-lasting depressive symptoms than previous generations in Germany, the rest of Europe and the USA (Hölzel et al., 2011). Furthermore, as somatic symptoms of depression are often underdiagnosed and undertreated, special consideration should be given to these specific symptoms (Greden, 2003). Consequently, more prevention and intervention efforts are needed as well as research on the potential reasons for the apparent increase across generations.

There are several possible explanations for this increase in depressive symptoms in younger birth cohorts in Germany. First, later born birth cohorts might be exposed to increasing mental health risks (Stephan et al., 2019). Situational characteristics relevant to depressive symptoms might have changed over time, such as increasingly stressful life circumstances, higher levels of social isolation and the use of social media (Beller & Wagner, 2018; Lin et al., 2016; Melchior et al., 2007). Additionally, habits are formed early in the life course and several studies have found that lifestyles differ according to one’s birth cohort (e.g., Macky et al., 2008). Thus, although empirical evidence is scarce, one potential explanation for the observed cohort differences might be that generations differ in important personal and situational characteristics like increasingly stressful environments and lower levels of physical activity.

Second, according to the expansion of morbidity and the dynamic equilibrium of morbidity hypotheses (Gruenberg, 1977; Manton, 1982), depressive symptoms might be on the rise because chronic diseases are also increasing. Depressive symptoms co-occur with other chronic diseases and impairments like diabetes and disability (Mukherjee & Chaturvedi, 2019; Noh et al., 2016): Depressive symptoms might be triggered as a psychological consequence of experiencing chronic physical disease; additionally, depressive symptoms and many physical diseases share common etiological pathways (Gold et al., 2020; Ogunmoroti et al., 2022). Furthermore, depressive symptoms are also found to be independent risk factors for many physical diseases (e.g., Dong et al., 2012; Knol et al., 2006). Thus, depressive symptoms might co-increase with associated chronic physical diseases. And indeed, multiple previous studies have found that conditions such as obesity, diabetes and disability are on the rise (Beller & Epping, 2020; Sperlich et al., 2020). As depressive symptoms represent a prevalent comorbidity of most chronic diseases, increasing rates of depressive symptoms could be partly explained by this rise of general morbidity among younger generations. This might also partly explain the observed relative stronger increases in somatic symptoms, because somatic depressive symptoms are most frequently found to co-occur with physical disease (Hays et al., 1998; Thom et al., 2019).

Lastly, in accordance with a “snowflake effect”, cognitive anchoring might affect survey responses. Perhaps, older cohorts have internalized other benchmarks of what it means to experience depressed affect, a lack of positive affect and depressive symptoms. If this was the case, the same levels of depressive symptoms might be rated as more prevalent by younger generations, although the “true” level of depression remains the same in the population. Lastly, future studies are needed to empirically examine the origin of these trends.

Countervailing effects of the time period were found by the current study, with comparatively smaller decreases in depressive symptoms across time period, as compared to the comparatively larger increases among younger birth cohorts. This result is in line with some previous studies, in which decreasing or stagnating trends in depressive symptoms over time were found, especially among older adults (Bretschneider et al., 2018; Kucera et al., 2020; Zivin et al., 2013) and mirrors the general trend toward more physically healthy aging often found in developed countries (Chatterji et al., 2015). The current study suggests that those prior findings might be caused by a synergetic effect of decreasing depressive symptoms across time periods and decreasing depressive symptoms for older birth cohorts born until about 1945. At the same time, numerous previous studies also suggested increasing trends in depressive symptoms across time in comparatively younger adults (e.g., Keyes et al., 2019). This likely reflects the increases in depressive symptoms among more recent born birth cohorts after 1945. However, as already discussed, the present study cannot estimate the causal reasons for the observed trends, which should be explored by future analyses.

Several limitations have to be taken into account when interpreting the results. The sample did not include institutionalized older adults and thus likely underestimates the true level of depression in the population. Similarly, I only studied depressive symptom trends in Germany, and future studies are needed to explore to what extent these results can be generalized to other European and international samples. Second, although I used one of the popular approaches to analysing birth cohort differences with the HAPC model, I acknowledge that there is a healthy discussion regarding the appropriate way to analyze age-period-cohort differences (Bell, 2020). Future studies might use other techniques suggested in the literature to validate the results (Fu, 2018; Yang & Land, 2013). Specifically, simulation studies suggest that the cohort effect might be underestimated by the methods used in the current study, which further emphasizes the need to validate the observed trends (Luo & Hodges, 2020). Similarly, age-period-cohort models cannot provide information on the causal reasons for the observed trends and future studies are needed that explicitly analyze potential explanatory factors for the observed generational rise in depressive symptoms. Finally, only a self-report measure of depression, the CES-D 15 was used. As discussed above, self-report measures might be susceptible to cognitive biases and as such future studies might employ other, potentially more objective indicators of depression (Marsden & Wright, 2010).

## Figures and Tables

**Fig. 5:**
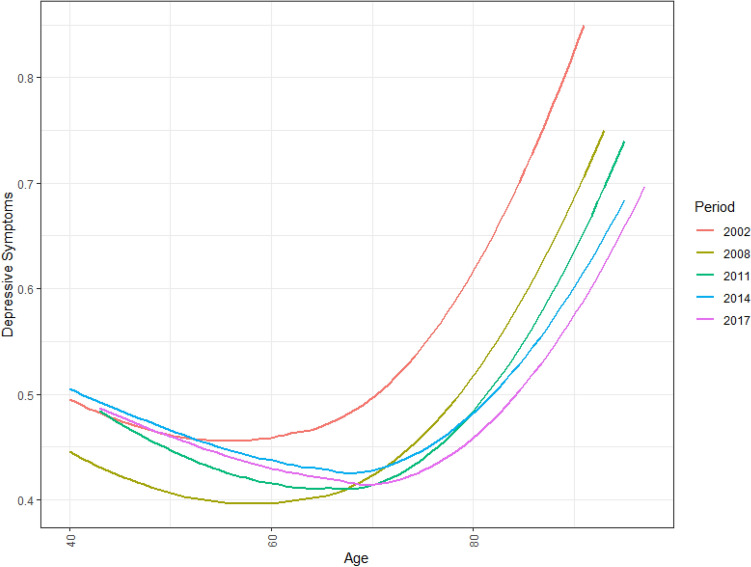
Depressive symptoms (mean-scored on a scale of 0–3) across age in different time periods

**Fig. 6:**
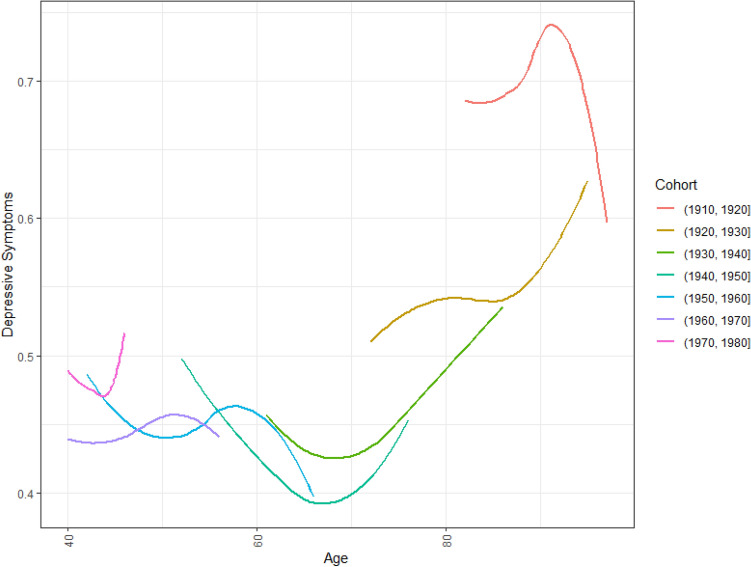
Depressive symptoms (mean-scored on a scale of 0–3) across age in different birth cohorts

**Fig. 7:**
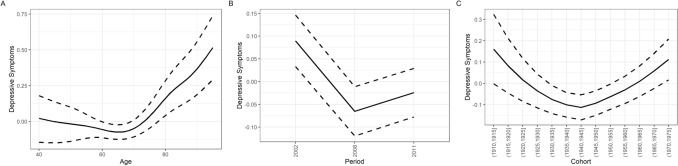
Age, period, and cohort differences (z-standardized) in depressive symptoms using only baseline first-time participants according to the HAPC model. Panel A depicts the predicted value of depressive symptoms across age; panel B depicts the predicted value of depressive symptoms across time periods; panel C depicts the predicted value of depressive symptoms across birth cohorts. Dashed lines indicate the 95% confidence intervals

**Fig. 8:**
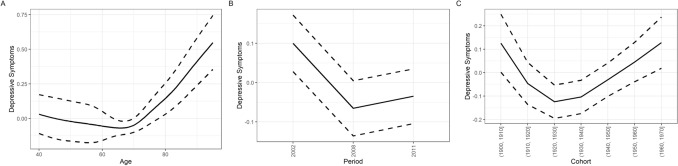
Age, period, and cohort differences (z-standardized) in depressive symptoms using a 10-year cohort operationalization according to the HAPC model. Panel A depicts the predicted value of depressive symptoms across age; panel B depicts the predicted value of depressive symptoms across time periods; panel C depicts the predicted value of depressive symptoms across birth cohorts. Dashed lines indicate the 95% confidence intervals

## Data Availability

The datasets supporting the conclusions of this article are available in the repository of the German Centre of Gerontology, https://www.dza.de/en/research/fdz/german-ageing-survey/data. However, restrictions apply to the availability of these data, which were used under license for the current study, and so are not publicly available.

## Appendix

See Figs. 5, 6, 7 and 8.

Tables 2, 3, 4 and 5

**Table 2:** Age-period-cohort analysis results predicting depressive symptoms

	*B/SD*	*95%-CI*	*χ*^*2*^	*p*
*Fixed effects*				
Age (centered)	0.0067	[0.0033; 0.0101]	15.1785	< .001
Age^2^ (centered)	0.0001	[0.0000; 0.0002]	6.8618	.009
*Random effects*				
Cohort	0.1143	[0.0631; 0.1837]	99.1852	< .001
Period	0.0663	[0.0327; 0.1421]	47.4240	< .001

*B* = Regression coefficient; *SD* = Random effects standard deviation; *95%-CI* = 95%-confidence interval; *χ*^*2*^ = χ^2^-Value; *p* = *p*-Value

**Table 3:** Age-period-cohort analysis results predicting depressed affect

	*B/SD*	*95%-CI*	*χ*^*2*^	*p*
*Fixed effects*				
Age (centered)	0.0031	[0.0000; 0.0063]	3.8126	.051
Age^2^ (centered)	0.0001	[0.0000; 0.0002]	3.1274	.077
*Random effects*				
Cohort	0.1020	[0.0492; 0.1716]	51.1045	< .001
Period	0.0657	[0.0314; 0.1398]	20.7680	< .001

*B* = Regression coefficient; *SD* = Random effects standard deviation; *95%-CI* = 95%-confidence interval; *χ*^*2*^ = χ^2^-Value; *p* = *p*-Value

**Table 4:** Age-period-cohort analysis results predicting positive affect

	*B/SD*	*95%-CI*	*χ*^*2*^	*p*
*Fixed effects*				
Age (centered)	0.0081	[0.0059; 0.0103]	51.8583	< .001
Age^2^ (centered)	0.0002	[0.0001; 0.0003]	13.4371	< .001
*Random effects*				
Cohort	0.0583	[0.0345; 0.0937]	69.2734	< .001
Period	0.0784	[0.0400; 0.1588]	45.4918	< .001

*B* = Regression coefficient; *SD* = Random effects standard deviation; *95%-CI* = 95%-confidence interval; *χ*^*2*^ = χ^2^-Value; *p* = *p*-Value

**Table 5:** Age-period-cohort analysis results predicting somatic symptoms

	*B/SD*	*95%-CI*	*χ*^*2*^	*p*
*Fixed effects*				
Age (centered)	0.0078	[0.0044; 0.0112]	20.0062	< .001
Age^2^ (centered)	0.0001	[0.0000; 0.0002]	5.7895	.016
*Random effects*				
Cohort	0.1217	[0.0664; 0.1933]	83.2202	< .001
Period	0.0508	[0.0257; 0.1113]	52.7778	< .001

*B* = Regression coefficient; *SD* = Random effects standard deviation; *95%-CI* = 95%-confidence interval; *χ*^*2*^ = χ^2^-Value; *p* = *p*-Value
